# Novel Photodynamic Therapy for Esophageal Squamous Cell Carcinoma following Radiotherapy

**DOI:** 10.3390/life13061276

**Published:** 2023-05-29

**Authors:** Takumi Yanagita, Takuto Hikichi, Jun Nakamura, Minami Hashimoto, Tsunetaka Kato, Rei Suzuki, Mitsuru Sugimoto, Yuki Sato, Hiroki Irie, Tadayuki Takagi, Masao Kobayakawa, Hiromasa Ohira

**Affiliations:** 1 Department of Endoscopy, Fukushima Medical University Hospital, Fukushima 960-1295, Japan; takumi-y@fmu.ac.jp (T.Y.); junn7971@fmu.ac.jp (J.N.); mi-hashi@fmu.ac.jp (M.H.); tsune-k@fmu.ac.jp (T.K.); mkobaya@fmu.ac.jp (M.K.); 2 Department of Gastroenterology, Fukushima Medical University School of Medicine, Fukushima 960-1295, Japan; subaru@fmu.ac.jp (R.S.); kita335@fmu.ac.jp (M.S.); dorcus@fmu.ac.jp (Y.S.); hirokiri@fmu.ac.jp (H.I.); daccho@fmu.ac.jp (T.T.); h-ohira@fmu.ac.jp (H.O.); 3 Medical Research Center, Fukushima Medical University, Fukushima 960-1295, Japan

**Keywords:** chemoradiotherapy, esophageal squamous cell carcinoma, local failure, photodynamic therapy, radiotherapy

## Abstract

Radiotherapy (RT) or chemoradiotherapy (CRT) are frequently selected as treatments for esophageal squamous cell carcinoma (ESCC). However, salvage treatment remains challenging when endoscopic resection is not indicated for residual or recurrent ESCC following RT or CRT. Recently, owing to the emergence of second-generation photodynamic therapy (PDT) using talaporfin sodium, PDT can be performed with less phototoxicity and therefore has regained popularity in the treatment of ESCC. In this study, the effectiveness and safety of second-generation PDT in patients with residual or recurrent ESCC following RT or CRT were examined. Local complete response (L-CR) rates, procedure-related adverse events, and prognosis were evaluated. In 12 patients with 20 ESCC lesions, the L-CR rates were 95.0%. Perforation, postoperative bleeding, and photosensitivity were not observed. Esophageal stricture following PDT developed in one patient, but this could be addressed using balloon dilation. During a median follow-up period of 12 (range, 3–42) months, the 3-year cause-specific survival rate was 85.7%. Even in patients with a Charlson comorbidity index score ≥ 3, the 2-year overall survival rates were 100%. In conclusion, PDT was an efficacious and a safe salvage treatment in patients with local residual or recurrent ESCC following RT or CRT.

## 1. Introduction

Esophageal squamous cell carcinoma (ESCC) is a malignant tumor that develops from the squamous cells lining the esophagus. Traditionally, esophagectomy has been the basic standard treatment option for ESCC.

Among treatments, chemoradiotherapy (CRT) has emerged as a potential treatment option for various stages of ESCC due to its ability to achieve a complete response (CR) and preserve the esophagus [1,2,3,4,5,6,7,8]. Studies have shown that the CR rates of CRT for ESCC were 87.5% for clinical stage I and 62.2% for clinical stages II–III [2,9]. In patients with early stage ESCC, the use of CRT is particularly advantageous, but in cases in which CRT is not feasible due to comorbidities, advanced age, or poor general condition, radiotherapy (RT) is used alone as a treatment option. Despite sufficient results reported for RT or CRT, recurrence or residual lesions remain major disadvantages [10,11]. Salvage therapy refers to additional treatment provided after RT or CRT has failed. Esophagectomy following RT or CRT is a curative treatment option for ESCC; however, it is a highly invasive procedure that is associated with high rates of postoperative complications and mortality when performed as a salvage treatment following RT or CRT [12]. Conversely, a minimally invasive treatment option for ESCC is endoscopic resection (ER). In particular, endoscopic submucosal dissection (ESD), which is mainly performed in East Asia, is a technique that can be used to achieve high en bloc resection rates. However, the indication for ESD is limited to intramucosal lesions. Therefore, submucosal (SM) invasion of the lesions or residual lesions after RT or CRT is not indicated for ESD. Furthermore, even if ESCC was an intramucosal lesion after RT or CRT, severe fibrosis was frequently found endoscopically or histopathologically [13]. This renders the removal of ESCC via ESD more challenging. Argon plasma coagulation (APC) is occasionally used as salvage treatment; however, APC is often less effective in cancers that have spread to the submucosa, and its use is restricted to lesions <20 mm [14].

Photodynamic therapy (PDT) employs a photosensitizer (PS) and an excitation laser tuned to the PS’s absorption wavelength. In this therapy, a cancer-selective PS is pre-administered, and an optimal wavelength laser light is irradiated around the cancer; this produces singlet oxygen in cancer cells. As a result, the effect of the singlet oxygen or the oxidative stress caused by reactive oxygen species from inflammatory cells induces apoptosis. The mechanism of apoptosis is said to vary depending on the PS. The hydrophilic PS mainly localizes to lysosomes in cancer cells; damage to caused lysosomes by PDT causes the cytokine cathepsin to be released from the lysosomes. In turn, this damages Blc-2, which is an apoptosis inhibitory protein that is present in the mitochondrial membrane and induces apoptosis. This induction and promotion of apoptosis occurs regardless of the cell cycle or genetic factors, such as p53, and is therefore effective in many organ tumors, regardless of tumor type [15,16]. PDT also ruptures tumor blood vessels and reduces blood flow to the tumor. The resulting hemorrhage and hypoxia have also been shown to cause tumor necrosis. This mechanism is also considered important [17]. Therefore, PDT is distinct from the necrosis induction in cancer cells caused by physical thermal transpiration using high-power lasers. In contrast, the laser irradiation of noncancerous tissue is less likely to induce cell death due to the low uptake of the PS. Furthermore, since the PS is not incorporated into the cell nucleus, the risk of DNA damage, mutation, or carcinogenesis in noncancerous cells is low [18].

First-generation PDT, which uses porfimer sodium (Pfizer Japan, Tokyo, Japan), was approved in 1994 and demonstrated a preferable outcome and safety profile in the analysis of a large number of patients and with a long-term follow-up period for local failure after definitive CRT for ESCC [19,20,21,22]. However, it required 4–6 weeks of prolonged light shielding. Recently, a second-generation PDT, which is performed using a diode laser and talaporfin sodium (Laserphyrin; Meiji Seika Pharma Co., Ltd., Tokyo, Japan) as a PS, was developed. Second-generation PDT is indicated for treating recurrent and residual ESCC after RT or CRT. In a clinical trial, the local complete response (L-CR) rate of a second-generation PDT was 88.5% [23]. Based on the trial results, since 2015, salvage PDT utilizing talaporfin sodium and a diode laser has been approved for insurance coverage in Japan for recurrent or residual ESCC following RT or CRT. It can be an effective alternative treatment, especially in patients for whom esophagectomy is difficult, such as the elderly or those with severe comorbidities, or in patients for whom ESD is difficult, such as those with severe fibrosis or suspected SM invasion. Despite the less-invasive nature of PDT, limited studies exist investigating its safety and effectiveness. Therefore, we conducted a study to evaluate the outcomes of PDT using talaporfin sodium and a diode laser as a salvage treatment for ESCC following RT or CRT.

## 2. Materials and Methods

### 2.1. Patients

This retrospective cohort study was conducted at a single center at Fukushima Medical University Hospital to investigate the use of PDT for treating ESCCs following RT or CRT. Patients who received treatment between April 2018 and September 2022 were included, and patient information data were collected retrospectively from electronic medical records and endoscopic databases from December 2022. In cases in which follow-up was performed at a different institution, the responsible physician contacted the patients to confirm their status and ascertain the cause of death from their families. 

The Declaration of Helsinki guidelines were followed in the conduct of this study, which was approved by the Institutional Ethics Committee of Fukushima Medical University (No. 2021-094, date of approval: 22 June 2021).

### 2.2. Practice of PDT and Follow-Up

The following inclusion criteria for second-generation PDT were established based on the criteria of the clinical trial of Yano et al. [23], which was based on first-generation PDT [19,20]. (1) Recurrence or residual ESCC pathologically confirmed in the area treated with RT or CRT. Both local and metachronous lesions were considered recurrence. Local recurrence was defined as lesions that recurred at the same site as the lesion that achieved CR via RT or CRT, and metachronous recurrence was defined as lesions that recurred at a site distant from the lesion that achieved CR via RT or CRT. (2) A depth of tumor invasion after RT or CRT that was limited to clinical T1–T2. The thickness and depth of the lesion were confirmed via endoscopic imaging and endoscopic ultrasonography (EUS) using a 20 MHz ultrasound probe. (3) The circumferentiality of the lesion was less than half of the esophagus. (4) Absent lymph node or distant metastasis, which was confirmed by computed tomography (CT) prior to PDT. (5) An inability to undergo or refusal for salvage surgery. (6) An inability to undergo or refusal for ER. Circumferentiality was chosen concerning stenosis and a decrease in the CR percentage. The wavelength of laser light used in second-generation PDT is 664 nm, which is longer than the 630 nm wavelength of the laser light used in first-generation PDT [19,20,23]. This allows the light to penetrate deeper into the tissue, allowing deeper areas to be treated than with first-generation PDT. As a result, the laser light is considered to reach into the muscle layer as far as the depth of the tumor [23].

Conversely, patients who satisfied the following exclusion criteria, which were developed based on the report of Yano et al., were not eligible for PDT [23]: (1) An Eastern Cooperative Oncology Group Performance Status of 3 or higher; (2) other active cancer requiring surgery or chemotherapy; and (3) aortic involvement before RT or CRT.

The PDT technique was employed using an EG-L590WR endoscope and a LASEREO endoscopic system (FUJIFILM Co., Tokyo, Japan). The PDT procedure was initiated through the intravenous administration of talaporfin sodium at a dose of 40 mg/m^2^, followed by laser irradiation at a 664 nm wavelength after 4–6 h. The diode laser was delivered through a frontal light distributor and the working channel of the endoscope, and a black-colored plastic attachment was fitted onto the scope tip to maintain a constant distance between the scope and the lesion. The fluence of the diode laser was set at 100 J/cm^2^ with a fluence rate of 150 mW/cm^2^. If the lesions were larger than 1 cm^2^, multiple treatment fields were overlapped to ensure full coverage of the entire lesion. One day post-treatment, endoscopic observation was mandatory, and additional laser irradiation was recommended if an obvious residual lesion was present. An obvious residual tumor was defined as the presence of a residual SM tumor-like protrusion, the presence of neoplastic mucosa or ulcer, or the absence of edematous mucosa with redness or dark blue discoloration resulting from incomplete irradiation. 

We conducted endoscopic follow-up examinations at intervals of at least 1, 2, and 8 weeks after PDT. A biopsy was conducted if a post-PDT remnant was suspected. If ESCC was diagnosed through biopsy, PDT was repeated in case the criteria were met. Lesions that were deemed to be deeper than the clinical SM at the time of PDT were followed up using CT scans every 3–6 months.

### 2.3. Outcomes

The efficacy of the PDT was assessed by analyzing the L-CR rates and the occurrence of procedure-related adverse events (aEs). Additionally, the overall survival (OS) and cause-specific survival (CSS), with OS and CSS being measured from the date of the first PDT to the date of death or the latest confirmation of survival, were evaluated. L-CR was defined as the absence of any endoscopic evidence of the lesion upon the disappearance of post-PDT ulcers. In cases in which PDT was repeated for the same lesion due to residual tissue, the local response was calculated using the best response. One session was defined as the day of treatment and the next day’s irradiation, and the number of sessions performed for each lesion and the required irradiation dose per session were examined. aEs were assessed and graded according to the Common Terminology Criteria for Adverse Events (CTCAE), version 5.0. Procedure-related aEs were classified as perforations, postoperative bleeding, and stenosis. Perforations were identified endoscopically as mediastinal or free air on CT scans when perforation was suspected during PDT. Postoperative bleeding was defined as the occurrence of hematemesis or tarry stools after PDT, as well as active bleeding or exposed blood vessels observed on endoscopy. Stenosis was defined as the presence of dysphagia symptoms and the inability of the 9-millimeter diameter endoscope to pass through the treated area after PDT. The PDT technique and the causes of aEs were retrospectively reviewed for each case. Death cases were also reviewed for their cause of death.

### 2.4. Statistical Analysis

The continuous variables of patient characteristics were presented as medians with ranges. Survivals were estimated using the Kaplan–Meier method and were analyzed using the log-rank test. Statistical significance was set at *p* < 0.05. The analyses were performed using EZR (Saitama Medical Center, Jichimu Medical University, Saitama, Japan), a graphical user interface for R (The R Foundation for Statistical Computing, Vienna, Austria) [24].

## 3. Results

### 3.1. Patient and Lesion Characteristics

A total of 20 ESCC lesions in 12 cases were identified, and all of these were included in the analysis (Table 1). Five patients had a Charlson comorbidity index (CCI) score of 3 or higher. Comorbidities included congestive heart failure in two patients and chronic lung disease, mild liver disease, diabetes mellitus, and moderate or severe liver disease in one patient each. Nine patients (75%) had received previous CRT, and three (25%) had received previous RT without chemotherapy. The T stages before RT/CRT were T1, T2, T3, and T4 in four (33.3%), three (25%), one (8.3%), and four patients (33.3%), respectively. The tumor statuses following RT/CRT were local recurrence, metachronous recurrence, and residual in five (41.7%), three (25%), and four patients (33.3%), respectively. The median time from the previous therapy to the index PDT in recurrent lesions was 46 (range, 16–39) months.

The characteristics of the 20 ESCC lesions are summarized in Table 2. The median tumor diameter was 15 mm (range, 5–40 mm). The lesion circumference of the esophageal lumen was 1/4 circumference of the esophagus in 14 lesions (70%), while one lesion was 1/2 circumference of the esophagus. The predicted depths before PDT were from the epithelium to the lamina propria mucosae, from the muscularis mucosae to the SM1, and from the SM2 to the SM3 in 11 (55%), three (15%), and six patients (30%), respectively. Among the nine lesions evaluated using EUS, none had an SM invasion length beyond 5.0 mm. One had an elevated lesion measuring approximately 10 mm; however, the SM invasion length was 4.5 mm.

### 3.2. PDT Outcomes

**Figure 1:**
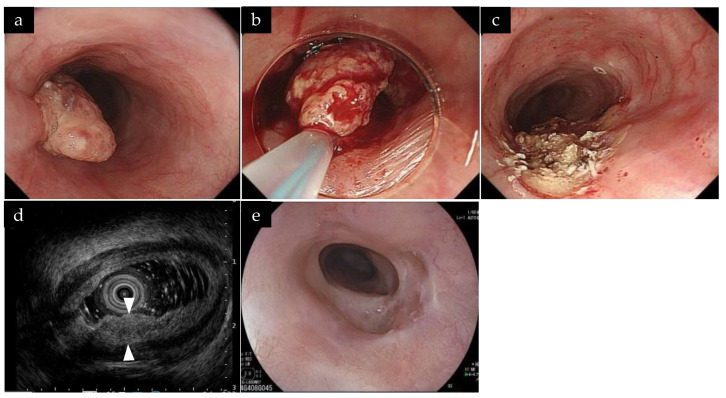
A case of a local recurrent lesion with a 10-millimeter-high elevated component. (**a**) A 10-millimeter-high elevated component was observed 1 year and 2 months after dCRT. (**b**) The elevated component was removed using a snare and high-frequency current without submucosal injection. (**c**) After ER, the longitudinal diameter was 30 mm, and the circumference was half the circumference of the esophageal lumen. (**d**) On EUS following the ER, the lesion thickness was 4.5 mm, and the cancer depth extended up to the deep submucosa. (**e**) Endoscopy conducted 1 year and 7 months after the initial PDT showed the scar, and no lesions or stenosis were observed. dCRT, definitive chemoradiotherapy; ER, endoscopic resection; EUS, endoscopic ultrasound; ESCC, esophageal squamous cell carcinoma; PDT, photodynamic therapy.

Eighteen lesions were treated in one session, and two lesions required two and three sessions. Of the 23 total sessions, 5 (21.7%) were irradiated on the next day, and the median total dose of irradiation was 345 (range, 64–800) J/lesion/session. One case of recurrent ESCC with a height of 10 mm was treated with PDT following the resection of the lesion in the ER to reduce the lesion height of the ESCC (Figure 1). This case has already been published as a case report [25].

The L-CR rate was 95.0% (95% confidence interval (CI): 74.6–100%) (Table 3). The median follow-up period was 12 months, with a range of 3 to 42 months. One patient died after the PDT. The lesion had a depth of SM2, and the lesion invasion length on EUS was 5.0 mm. The patient did not provide consent for esophagectomy. Therefore, two sessions of PDT were performed, but did not result in L-CR. Later, esophagectomy was performed due to the appearance of lymph node metastasis. Three months after esophagectomy, multiple liver and lung metastases appeared. Unfortunately, he died from ESCC 11 months after the initial PDT. This was the only ESCC death case, and no ESCC deaths were observed in patients who achieved L-CR after PDT.

**Figure 2:**
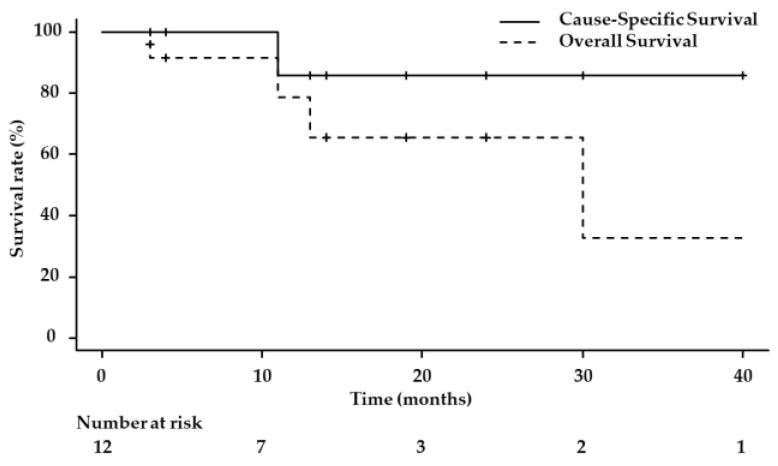
Overall and cause-specific survival rates. The 1-year overall and cause-specific survival rates were 78.6% and 85.7%, respectively. The 3-year overall and cause-specific survival rates were 32.7% and 85.7%, respectively.

**Figure 3:**
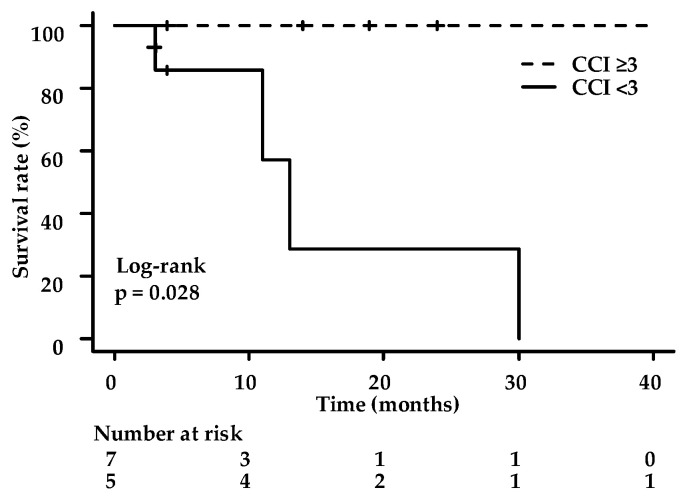
Overall survival rates with low CCI (<3) or high CCI scores (≥3). The 1-year overall survival rates with the low- and high-CCI-score groups were 57.1% and 100%, respectively. The 2-year overall and cause-specific survival rates were 28.6% and 100%, respectively. CCI, Charlson comorbidity index.

The 1-year OS rate was 78.6%, and the 1-year CSS rate was 85.7%, while the 2-year OS and CSS rates were 65.5% and 85.7%, respectively (Figure 2). During the follow-up period, four patients (33.3%) died from other causes, including tongue cancer, ureteral cancer, brain tumor, and an unknown cause. Furthermore, the study analyzed factors associated with OS and found that patients with CCI scores of 3 or higher had higher OS rates (*p* = 0.028, Figure 3).

**Figure 4:**
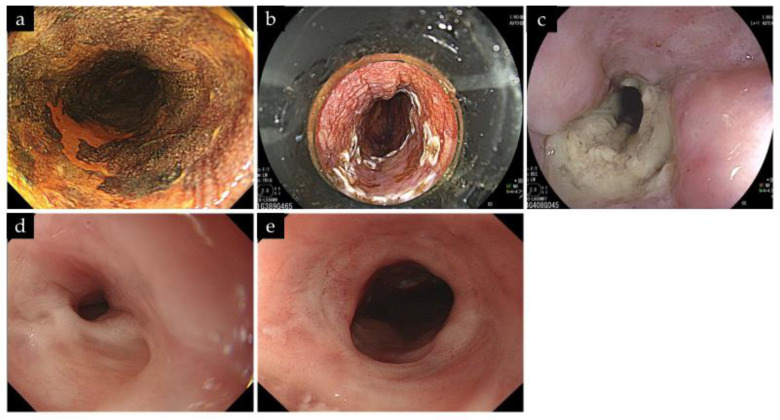
A case of esophageal stricture after PDT. (**a**) A recurrent ESCC with a size of 15 mm and 1/3 circumference was observed in the middle thoracic esophagus. (**b**) The plastic attachment was fitted in front of the scope to maintain the distance between the tip of the scope and lesion. Multiple treatment fields were overlapped to cover the entire lesion. The total irradiation dose was 318 J without irradiation on the next day. (**c**) Endoscopic image at 1 month after initial PDT showed a circumferential ulcer. (**d**) Endoscopic image at 6 months after initial PDT showed an esophageal stricture. The endoscope failed to pass through. (**e**) After four balloon dilatations with local steroid injections, esophageal stricture improved at 1 year after initial PDT. ESCC, esophageal squamous cell carcinoma; PDT, photodynamic therapy.

A summary of PDT-related aEs is shown in Table 4. Perforation, postoperative bleeding, or photosensitivity were not observed. The most frequent AE was chest pain, occurring in 5 out of 23 sessions. One patient had CTCAE grade 3 esophageal stenosis. This case could be treated with a total of four balloon dilatations combined with local steroid injections. No restenosis was observed for more than 1 year after the initial PDT (Figure 4). 

## 4. Discussion

In the present study, PDT was an effective salvage therapy in patients with ESCC following RT or CRT; the 3-year CSS rate in all patients was 85.7%. Furthermore, PDT was demonstrated to be safe for patients with several comorbidities, including those with CCI scores of ≥3. Although this was a single-center study, a detailed retrospective examination of the procedural innovations, aEs, and prognosis for each case was conducted, which is one of the strengths of our study.

ER or esophagectomy for patients with ESCC following RT or CRT is frequently considered challenging owing to several factors, including adhesions, fibrosis, comorbidity, and aging. PDT using porfimer sodium and excimer dye laser in cases of residual or recurrent ESCC was reported by Yano et al. [26]. However, PDT using porfimer sodium requires a long light-shielding period of 4 weeks. Yano et al. developed PDT using talaporfin sodium and a diode laser [23]. The L-CR rates following talaporfin PDT in ESCC following RT or CRT have been reported to be 69–88.8% in single-center studies [27,28,29,30,31,32]. In this study, the L-CR rate was 95.0% (95% CI: 74.6–100%), showing a relatively higher L-CR rate than those of previous reports. 

Mitsui et al. reported that EUS findings, including tumor thickness <5.0 mm or tumor invasion length into the muscularis propria layer <1.6 mm, were significantly associated with achieving L-CR following PDT [33]. In this study, the high L-CR rate may be attributed to the absence of T2 lesions; although T2 lesions were included in the PDT criteria, there were no applicable cases in this study. In our study, in one case, the presence of a 10 mm elevated lesion made it difficult to achieve a cure through PDT alone [25]. Due to the elevation component, the SM invasion evaluation was also difficult. Initially, the elevated component was removed using an ER technique. Subsequently, EUS revealed that the SM invasion depth was less than 5 mm. PDT was then performed, and L-CR was achieved. If the elevated component had been left, the laser light would not have reached the submucosa, making it impossible to achieve L-CR. Combination therapy of PDT with ER was suggested to be effective for an ESCC with a high elevation component. 

Conversely, one patient who did not achieve L-CR had a tumor thickness of 5.0 mm, observed via EUS. This was the thickest lesion among the nine lesions whose thickness could be measured via EUS. One of the rationales for why L-CR was not achieved in this case was that the laser light failed to reach sufficiently deep to render an anti-tumor effect. When PDT is selected as a salvage therapy, esophagectomy is often not possible for some reason. However, this case showed that esophagectomy should be reconsidered as a treatment option when L-CR cannot be achieved in a single session in a thick lesion. Furthermore, if L-CR is not achieved, lymph node metastasis may occur before the ulcer heals after PDT; hence, an imaging evaluation such as CT or endoscopy is important for thick lesions or lesions with a high risk of metastasis.

In one patient, PDT caused esophageal stricture. This was because the postoperative ulcer was almost entirely circumscribed. In retrospect, we considered that the laser had reached the opposite wall beyond the black-colored plastic attachment, unless the lesion was small. This may have been attributed to the lack of skill of the operators; therefore, improving the skill of the operators is important. In this patient, endoscopic balloon dilation was an effective treatment option for esophageal stricture following salvage PDT. Intralesional steroid injection or systemic steroid therapy has been reported to prevent stricture following ESD for ESCC [34,35,36]. However, owing to the residual tumor, local steroid injection immediately after PDT irradiation is impractical. This case was successfully treated with balloon dilatation in combination with the local injection of steroids for the stenotic area after confirmation of L-CR. Systemic steroid administration may be initiated immediately after irradiation. However, to our knowledge, to date, systemic steroid therapy for the prevention of esophageal stricture following PDT has not been studied. Further studies are warranted on the prevention and treatment of stricture.

Even in patients with CCI ≥ 3, the OS following PDT was longer than those with CCI < 3. Therefore, PDT is an effective palliative therapy for patients with multiple comorbidities as well. Although the CCI score has been shown to be a prognostic factor for patients with ESCC [37,38,39], the preoperative CCI value was insufficient for predicting postoperative outcomes. This is because postoperative malignancy variables not related to the preoperative CCI value became the main cause of death. Patients with ESCC have a high probability of having two or more primary lesions owing to their long-term smoking and drinking habits and advanced age. In this study, the patient background also had a high rate of alcohol consumption and smoking and a high risk of carcinomas other than esophageal cancer. PDT was performed on patients without other cancers before treatment; however, the other cancer became the main cause of death. To extend the prognosis of patients who will receive salvage PDT, performing cancer surveillance of other organs is imperative.

As described above, compared to the first-generation PDT using porfimer sodium, the second-generation PDT using talaporfin sodium is expected to have a higher antitumor effect on residual or recurrent ESCC after RT or CRT. Another advantage of the second-generation PDT is that the long light-shielding period of 4 weeks, which was a major issue with the first-generation PDT, lasts only about 1 week with the second-generation PDT. As a result, no photosensitivity developed in this patient group. The cost of the laser generator is also low, costing JPY 9 million, compared with the cost of JPY 45 million for the first-generation PDT. However, a vial of talaporfin sodium is expensive, costing JPY 340,000 per vial.

This study had several limitations. First, it was a single-center retrospective study. However, we consider the strength of this paper to be the fact that the single-center study allowed us to examine each case in retrospective detail. Second, the sample size was small, and the follow-up period was limited. In particular, no case of clinical T2 lesions was observed. It is necessary to confirm the factors associated with the OS of PDT in a larger number of patients and with longer follow-up periods. However, the safety and effectiveness of PDT for patients with high CCI scores could be demonstrated even in a small number of cases.

## 5. Conclusions

The application of PDT for ESCC patients after undergoing RT or CRT is a promising and safe option for preventing mortality caused by ESCC. However, further investigations should be conducted to assess the efficacy of PDT for ESCC following RT/CRT in a larger cohort of patients at different medical centers and with a longer duration of follow-up.

## Figures and Tables

**Table 1:** Patient characteristics (n = 12).

Patients/lesions, n	12/20
Age, median (range)	78 (71–87)
Sex, male/female	11/1
Height, median (range), m	1.60 (1.52–1.73)
Weight, median (range), kg	59.9 (43.4–83.8)
Body mass index, median (range)	23.3 (18.1–32.7)
Smoking status	
Current or former smoker, n (%)	8 (66.7)
Never smoked, n (%)	4 (33.3)
Alcohol status	
Current or former drinker, n (%)	12 (100)
Never drank, n (%)	0
ECOG performance status, n (%)	
0	8 (66.7)
1	4 (33.3)
Intake of antithrombotics, n (%)	2 (16.7)
Comorbidity, n (%)	
Congestive heart failure	2 (16.7)
Chronic lung disease	1 (8.3)
Mild liver disease	1 (8.3)
Diabetes	1 (8.3)
Moderate or severe liver disease	1 (8.3)
Charlson comorbidity index score, n (%)	
<3	7 (58.3)
≥3	5 (41.7)
T stage before RT/CRT, n (%)	
T1	4 (33.3)
T2	3 (25)
T3	1 (8.3)
T4	4 (33.3)
Lymph node metastases before RT or CRT on CT, n (%)	
Absent	10 (83.3)
Present	2 (16.7)
Prior treatment	
CRT	9 (75)
RT alone	3 (25)
Total radiotherapy dose (Gy), n (%)	
≤60	8 (66.7)
>60	4 (33.3)
Lesion status following RT or CRT, n (%)	
Local recurrence	5 (41.7)
Metachronous recurrence	3 (25)
Residual	4 (33.3)
Recurrence period from previous treatment to index PDT, median (range), month	46 (16–39)

CRT, chemoradiotherapy; CT, computed tomography; ECOG, Eastern Cooperative Oncology Group; PDT, photodynamic therapy; RT, radiotherapy.

**Table 2:** Lesion characteristics (n = 20).

Location, n (%)	
Upper thoracic esophagus	4 (20)
Middle thoracic esophagus	14 (70)
Lower thoracic esophagus	2 (10)
Abdominal esophagus	0
Tumor diameter, median (range), mm	15 (5–40)
Circumference, n (%)	
<1/4	14 (70)
≥1/4, <1/2	5 (25)
≥1/2	1 (5)
Lesion thickness in EUS, median (range), mm	2.5 (0–5.0)
Predicted depth before PDT	
EP–LPM	11 (55)
MM–SM1	3 (15)
SM2–SM3	6 (30)

EP, epithelium; EUS, endoscopic ultrasound; LPM, lamina propria mucosae; MM, muscularis mucosae; SM, submucosae; PDT, photodynamic therapy.

**Table 3:** Clinical outcomes following photodynamic therapy.

L-CR, % (n)	95.0 (19/20)
Death by esophageal squamous cell carcinoma *, n (%)	8.3 (1/12)
Death by other disease **, n (%)	33.3 (4/12)
Follow-up period median (range) months	12 (3–42)

L-CR, local complete response; ESCC, esophageal squamous cell carcinoma; * The dead patient is a non-complete response case. ** Tongue cancer, ureteral cancer, brain tumor, and unknown-cause cases.

**Table 4:** Adverse events related to photodynamic therapy (n = 23).

Procedure-Related Adverse Events, n	
Poststernal chest pain	5 (21.7)
Esophageal stenosis	1 (4.3)
Fever	1 (4.3)
Pneumonia	0
Delirium	0
Esophageal hemorrhage	0
Esophageal perforation	0
Skin phototoxicity	0
Grade ≥ 3 adverse events *	1 (4.3)

* Esophageal stenosis.

## Data Availability

Data are available upon request due to restrictions, for example, privacy or ethical considerations.
